# Big Data analytics for improved prediction of ligand binding and conformational selection

**DOI:** 10.3389/fmolb.2022.953984

**Published:** 2023-01-12

**Authors:** Shivangi Gupta, Jerome Baudry, Vineetha Menon

**Affiliations:** ^1^ Department of Computer Science, The University of Alabama in Huntsville, Huntsville, AL, United States; ^2^ Department of Biological Sciences, The University of Alabama in Huntsville, Huntsville, AL, United States

**Keywords:** protein conformation selection, Big Data, deep learning, machine learning, feature selection, drug discovery

## Abstract

This research introduces new machine learning and deep learning approaches, collectively referred to as Big Data analytics techniques that are unique to address the protein conformational selection mechanism for protein:ligands complexes. The novel Big Data analytics techniques presented in this work enables efficient data processing of a large number of protein:ligand complexes, and provides better identification of specific protein properties that are responsible for a high probability of correct prediction of protein:ligand binding. The GPCR proteins ADORA2A (Adenosine A2a Receptor), ADRB2 (Adrenoceptor Beta 2), OPRD1 (Opioid receptor Delta 1) and OPRK1 (Opioid Receptor Kappa 1) are examined in this study using Big Data analytics techniques, which can efficiently process a huge ensemble of protein conformations, and significantly enhance the prediction of binding protein conformation (i.e., the protein conformations that will be selected by the ligands for binding) about 10–38 times better than its random selection counterpart for protein conformation selection. In addition to providing a Big Data approach to the conformational selection mechanism, this also opens the door to the systematic identification of such “binding conformations” for proteins. The physico-chemical features that are useful in predicting the “binding conformations” are largely, but not entirely, shared among the test proteins, indicating that the biophysical properties that drive the conformation selection mechanism may, to an extent, be protein-specific for the protein properties used in this work.

## 1 Introduction

The prediction of which small molecules, e.g., substrates or modulators, are more likely than other small molecules to bind to a specific protein, is one the most formidable challenges of contemporary biology, chemical biology and pharmacology. Only a small fraction of the large number of small organic molecules present in living organisms will, in most cases, bind to a specific protein. There is a considerable amount of work that aims at improving the biophysical approaches to predicting such protein:ligand interactions.

As exemplified in the current special issue of Frontiers, the dynamics of the protein target is increasingly taken into account in such predictive approaches. Indeed, a protein cycles through multiple conformations, a few of which will be bound by its ligands, as conceptualized in the “conformational selection” mechanism of ligand binding. Virtual docking (Amaro et al., 2018) that aims at predicting if a given small chemical binds to a given protein, usually considers only one protein conformation in an “induced fit” mechanism. Advances beyond a simple induced fit mechanisms have been proposed, such as submitting the protein:ligands complexes to molecular dynamics simulations after docking (Seelinger and de Groot, 2010), which identifies binding modes of known ligands close to that of their experimental co-crystallized structures, or generating an ensemble of holo structures from experimental structures deposited in the PDB for a given protein target (Aggarwal et al., 2021). This present work, continuing in that direction, aims at using the information contained in molecular dynamics simulations of a single protein target structure prior to any docking.

In principle the “binding” protein conformations will correspond to the free energy minima of the (protein + ligand) complex free energy hypersurface. In our research, we are looking into whether we can identify these rare apo-conformations that possess this capacity to bind their ligands, while the vast majority of the other apo-protein conformations do not. This paper describes our Big Data analytics work toward such characterization of what properties of an apo-protein conformation more likely lead to conformational selection.

The data we used here has been obtained using supermassive “ensemble docking” from proteins’ molecular dynamics simulations, and is described in (Evangelista et al., 2016). The data corresponds to about 1.5 millions of protein conformation and protein:ligand complex structures and their associated docking scores.

Big Data analytics provides an efficient approach to analyzing such a large amount of data, and also addresses the class imbalance problem (Abd Elrahman and Abraham, 2013), which is a result of imbalanced groups or sub-categories present in the data, where the majority class or larger group of data consists of non-binding protein conformations and it overshadows the minority class or smaller data group, which comprises the data-of-interest i.e., the binding protein conformations. In our prior work (Akondi et al., 2019; Gupta et al., 2022; Sripriya Akondi et al., 2022), a novel two-stage sampling-based classifier framework was proposed with the primary goal of addressing the class imbalance problem and maximizing the detection of potential binding protein conformations as conventional machine learning (ML) algorithms are ill-equipped to deal with the issue of class imbalance during the data-learning phase. This paper extends on our previous work by presenting additional improvements to our two-stage sampling-based classification approach (Gupta et al., 2022) using deep learning techniques and four different feature selection methods in conjunction with an Enrichment ratio framework.

## 2 Materials and methods

### 2.1 Dataset description

As described in our previous work (Gupta et al., 2022), Molecular Dynamics (MD) simulations of four proteins, namely, ADORA2A (Adenosine A2a Receptor), ADRB2 (Adrenoceptor Beta 2), OPRD1 (Delta Opioid Receptor) and OPRK1 (Opioid Receptor Kappa 1) were used to study the efficacy of our proposed method. The conformations of these four proteins have been well-studied, and the protein conformations that: a) will bind to ligands (binding conformations) and b) will not bind to ligands (non-binding conformations), are known and have been previously documented and published (Evangelista et al., 2016).

ADORA2A: This dataset has 50 attributes and consists of 2,998 protein conformations among which 851 protein conformations are “binding” and 2,147 protein conformations that are “non-binding”. Here the imbalance ratio is 3:1 i.e., for every datasample belonging to minority class (binding conformations) there are three data samples belonging to the majority class (non-binding conformations).

ADRB2: This dataset has 51 attributes and consists of 2,565 protein conformations among which 156 are binding and 2,411 protein conformations are non-binding. Here the imbalance ratio is 16:1 i.e., for every datasample belonging to minority class (binding conformations) there are 16 data samples belonging to the majority class (non-binding conformations).

OPRD1: This dataset has 51 attributes and consists of 3,004 protein conformations among which 72 protein conformations are binding and 2,932 protein conformations are non-binding. Here the imbalance ratio is 41:1 i.e., for every datasample belonging to minority class (binding conformations) there are 41 data samples belonging to the majority class (non-binding conformations).

OPRK1: This dataset has 50 attributes and consists of 2,998 protein conformations among which 138 protein conformations are binding and 2,862 protein conformations are non-binding. Here the imbalance ratio is 20:1 i.e., for every data sample belonging to minority class (binding conformations) there are 20 data samples belonging to the majority class (non-binding conformations).

Tables describing the protein attributes/features/descriptors for ADORA2A, ADRB2, OPRD1, and OPRK1 datasets can be found in our previous work (Gupta et al., 2022). ADRB2 and OPRD1 have one additional feature - pro_pl_seq (Sequence based pI) in comparison to ADORA2A and OPRK1. The molecular descriptors were calculated using the protein descriptors from the program MOE (Akondi et al., 2019; Chemical Computing Group, 2019; Gupta et al., 2022; Sripriya Akondi et al., 2022).

#### 2.1.1 Analysis of variance

Analysis of variance (ANOVA) is a statistical analysis method used here to calculate the linear relationship between the various protein features and to select the important protein features that correspond to the highest F-values (Johnson and Synovec, 2002). The top “*x*” features with the greatest F-values were selected in this case, where the *x* features to be retained is determined experimentally by the user. Thus, ANOVA technique allows for selection of the primary physio-chemical protein properties that essay a critical role in protein:ligand interaction and conformation selection.

#### 2.1.2 Mutual information

Mutual Information (MI) (Macedo et al., 2019) is a measure of the amount of information that can be inferred about a variable **U** through the use of the other given random variable **V**. The mutual information **I** (**U**; **V**) for random variables **U** and **V** can be defined as follows (Guyon and Elisseeff, 2003; Gupta et al., 2022):

$$I\left(U;V\right)=-\underset{v\in V}{\sum }\underset{u\in U}{\sum }p\left(u,v\right)\log\frac{p\left(u,v\right)}{p\left(u\right)\;p\left(v\right)}$$ (1)

where

• p(u,v) is the joint probability density function.
• p(u) is the probability density function

In Eq. 1, if the MI value **I** is 1, then **U** and **V** are dependent on each other, i.e., protein features share similar information. If the MI value **I** is 0, then **U** and **V** are independent of each other i.e., no common (in other words unique) information between the features. The MI in physio-chemical properties are calculated as follows:

• First, calculate the MI value for all properties to determine how dependent the physio-chemical features vectors are and understand the common information contained in all the protein features.
• Then, sort the protein features according to their highest MI values. The top “*x*” protein features with the greatest MI values are retained, where *x* is user defined.

#### 2.1.3 Recurrence quantification analysis

Recurrence Quantification Analysis (RQA) is a non-linear data-analysis method that is used to study the dynamical systems (Eckmann et al., 1987). The first step in the recurrence analysis is to quantify the repeating patterns of a dynamic system. One of the variables generated by the quantification of the recurrences is Entropy (ENT), which is the probability distribution p(j) of the diagonal line on the RQA plot and is defined as:

$$ENTR=-\overset{M}{\underset{j=j_{\min}}{\sum }}p\left(j\right)\;\ln\left(p\left(j\right)\right)$$ (2)

where M is the number of points on the state space trajectory and j is the length of the diagonal line in the RQA plot. We investigate the RQA-based entropy measure’s link to the probability of detecting potential binding conformations in terms of time-space evolution of protein conformations.

#### 2.1.4 Spearman correlation coefficient

Spearman correlation coefficient is a statistical measure (Hauke and Kossowski, 2011) of the strength and direction of the monotonic relationship between each protein feature and target variable. The correlation coefficient for each feature is obtained by applying the formula as defined below:

$$\rho =\frac{\Sigma_{i}(u_{i}-\overset{-}{u})(v_{i}-\overset{-}{v})}{\sqrt{\Sigma_{i}{(u_{i}-\overset{-}{u})}^{2}{(v_{i}-\overset{-}{v})}^{2}}}$$ (3)

where u is the feature vector and $\overset{-}{u}$ is its corresponding mean. Similarly, v is the target vector and $\overset{-}{v}$ is the mean of the target vector. The Spearman correlation coefficients for protein features are computed, sorted and ranked based on the absolute value of the correlation coefficient. A subset of the protein features were then selected based on the “*x*” highest rankings, where *x* is user-defined. Therefore, the Spearman correlation coefficient allows us to select protein features that are strongly correlated with each other.

#### 2.1.5 Extreme gradient boosting

Extreme Gradient Boosting (XGBoost) is a tree ensemble boosting approach that merges a number of weak classifiers into a single strong classifier (Chen and Guestrin, 2016). Starting with a base learner, the strong learner is trained iteratively for best classification or prediction performance. Given a dataset X with *m* samples and *n* protein descriptors, let ( $\left.x_{1\;},y_{1}),...,(x_{k\;},y_{k}\right)$ be a set of inputs *x*_i_ and corresponding outputs y _i_ (Babajide Mustapha and Saeed, 2016). The XGBoost algorithm uses “K” additive functions, each representing a classification and regression tree (CART) to predict the output label $\hat{{\;y}_{i}}$ as defined by:

$$\hat{{\;y}_{i}}=\overset{K}{\underset{k=1}{\sum }}t_{k}\left(x_{i}\right),\;t_{k}\in T$$ (4)

where $t_{k}$ corresponds to a distinct tree structure with leaf score “w” and T is the space of all classification and regression trees. The goal is to minimize the following regularized objective function (Babajide Mustapha and Saeed, 2016):

$$Obj\left(\Theta \right)=\overset{m}{\underset{i}{\sum }}\;l\left(y_{i}\;,\;\hat{{\;y}_{i}}\right)+\overset{K}{\underset{k}{\sum }}\;\Psi \left(t_{k}\right)$$ (5)

where l is the loss function that is used to measure the difference between the predicted value $\hat{{\;y}_{i}}$ and the actual value $y_{i}\;$ and Ψ is the regularization term that is used to avoid overfitting and is defined as:

$$\Psi \left(t_{k}\right)=\gamma D+\frac{1}{2}\lambda {\left\|w\right\|}^{2}$$ (6)

where D is the number of leaves, w is the weight of each leaf, γ and λ are constants to control the degree of regularization.

#### 2.1.6 K-Means clustering

K-Means clustering is an unsupervised machine learning algorithm (Oyelade et al., 2010) that is used to understand the data patterns in the input data by grouping the instances in the dataset that are similar into different clusters. K-Means clustering is often used to produce compact clusters with minimum intra-cluster distances and maximum inter-cluster distances (Oyelade et al., 2010). This goal is achieved by splitting the data into a number of clusters “k” that the user specifies (Wilkin and Huang, 2007). Here we employ the K-Means clustering algorithm to under sample the data points from the majority class samples i.e., non-binding protein conformations as demonstrated in our prior work (Akondi et al., 2019).

#### 2.1.7 Generative adversarial networks

Generative adversarial networks (GAN) is an unsupervised learning method that involves learning regularities or patterns in the input data to produce new examples that mimic the original dataset. The GAN technique uses two artificial models, the discriminator and generator, which compete for data learning (Jo and Kim, 2022). The discriminator focuses on discriminating or distinguishing between the original and synthetic data, whereas the generator tries to create synthetic data that is comparable to the real data. The loss function of GAN (Jo and Kim, 2022) is defined as:

$$\min_{D}\max_{F}V\left(F,D\right)=Q_{u∼p_{u}}\left[\log\;\left(F\left(u\right)\right.\right]-Q_{v∼p_{v}}\left[\log\left(1-\left(F\left(D\left(v\right)\right)\right)\right.\right]$$ (7)

where.

• $p_{u}$ is the data-generating distribution
• $p_{v}$ is the noise distribution
• u is the real input data
• v is the noise input to the generator neural network
• F(u) is the output probability of the generator
• D(v) is the sample generated by the generator neural network

Here the GAN is used to oversample or replicate the minority class in the dataset to alleviate the class imbalance problem and in turn maximize the prediction of the potential binding protein conformations.

#### 2.1.8 Convolutional neural networks

**FIGURE 1:**
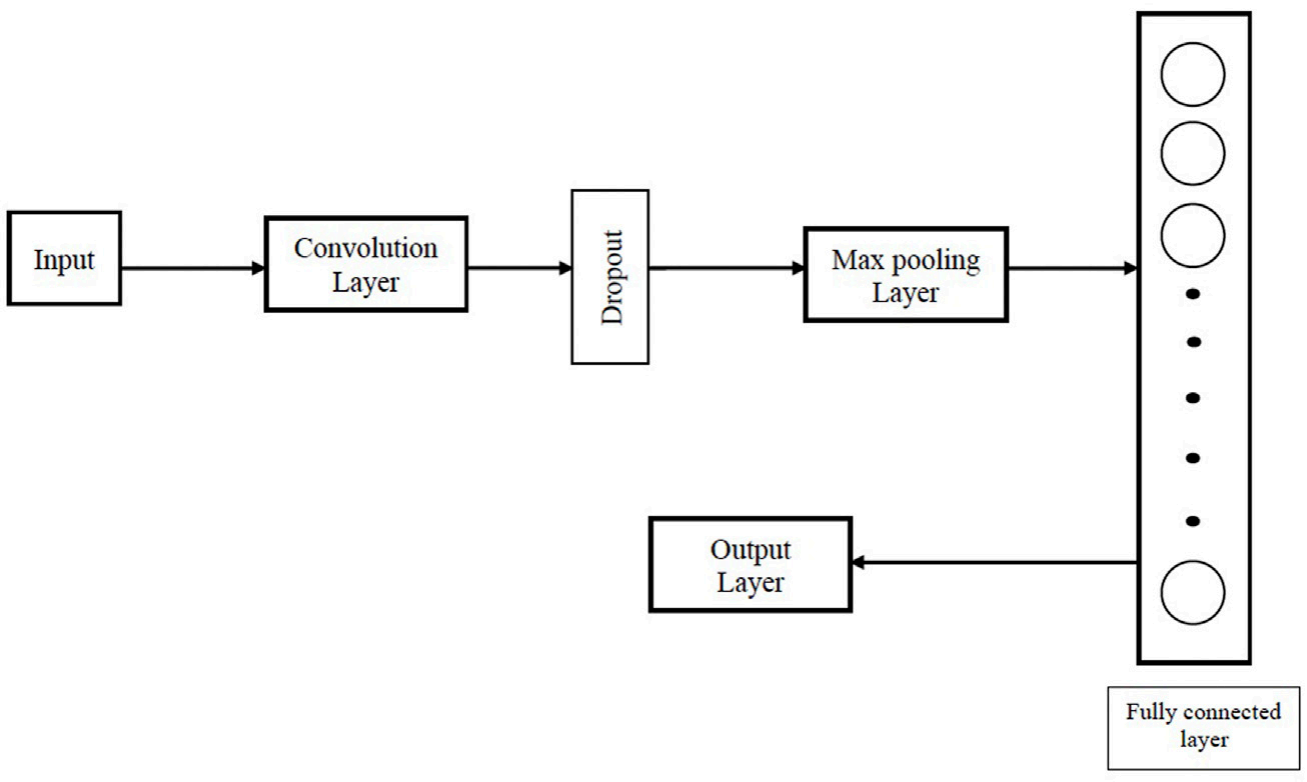
Architecture of the CNN used in our proposed Big Data analytics based AI/ML protein conformation selection/prediction framework.

Convolutional neural network (CNN) is a supervised deep learning technique (Hossain and Sajib, 2019) that has emerged as the most widely used artificial neural network in many computer vision applications, including texture recognition (Cimpoi et al., 2016), remote sensing scene classification (Hu et al., 2015; Penatti et al., 2015) and structure-based protein analysis (Torng and Altman, 2017). Architectural design of a CNN consists of several convolutional, pooling and dropout layers followed by one or more fully-connected layers (FC) (Sultana et al., 2018). Figure 1 describes the architecture of the CNN used in our work.

The architectural design of the CNN in our work consists of a convolutional layer followed by dropout to reduce overfitting, a max pooling layer, a fully connected layer and an output layer. Rectified linear unit (ReLU) is used as the activation function for the convolution layer and fully connected layer. Binary cross-entropy L is used as the loss function for the CNN.

#### 2.1.9 Recurrent neural networks

**FIGURE 2:**
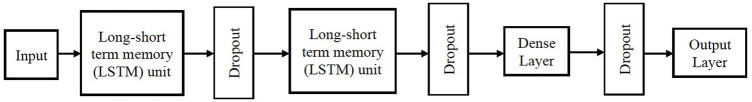
Architecture of the RNN used in our proposed Big Data analytics based AI/ML protein conformation selection/prediction framework.

Recurrent neural network (RNN) is a class of neural networks which is used to detect patterns in a sequence of data (Ho and Wookey, 2020). In our work, the RNN architecture consists of two long-short term memory (LSTM) (Schmidt, 2019) layers with dropout followed by a dense layer with dropout and an output layer. The LSTM unit introduces a gate mechanism to select whether to retain or discard specific information in the existing memory. If the LSTM unit recognizes a pivotal protein descriptor from an input sequence early on, then it captures any potential long-distance dependencies between the protein descriptor and target value. Figure 2 describes the architecture of the RNN used in our work. Rectified linear unit (ReLU) is used as the activation function for the LSTM unit and dense layer, sigmoid function is used as the activation function in the output layer and binary cross-entropy as the loss function.

#### 2.1.10 Evaluation metrics

The confusion matrix and its derived evaluation parameters such as classification accuracy, sensitivity, specificity, etc., are some of the most commonly used ML evaluation metrics to validate a classification or prediction performance of ML algorithms. In this case of binary classification between binding and non-binding protein conformations, the confusion matrix has four categories of classification results as follows:

• True Positive (TP): When the classifier accurately predicts “binding,” indicating that the ligand and target protein did bind (Right predictions of class 1)
• True Negative (TN): When the classifier accurately predicts “non-binding,” indicating that the ligand and target protein did not bind (Right predictions of class 0)
• False Negative (FN): When the classifier inaccurately predicted “non-binding,” but the ligand and target protein did bind (Wrong predictions of class 0)
• False Positive (FP): When the classifier inaccurately predicted “binding,” but the ligand and target protein did not bind (Wrong predictions of class 1)

Here class 0 refers to the non-binding protein conformations (majority class) and class 1 denotes the binding protein conformations (minority class).

Accuracy of an AI/ML framework is calculated as the sum of correctly predicted binding and non-binding protein conformations divided by the total number of conformations in the data set. It is defined as:

$$Accuracy=\frac{TP+FN}{TP+FP+FN+TN}$$ (8)

Sensitivity is the ability of the AI/ML framework to correctly predict binding protein conformations. It is calculated as the number of correctly predicted binding protein conformations divided by the total number of binding protein conformations in the data set as defined below:

$$Sensitivity=\frac{TP}{TP+FN\;}$$ (9)

Eqs 8, 9 are used for performance evaluation of the proposed AI/ML protein conformation selection/prediction framework.

#### 2.1.11 Enrichment ratio framework

The enrichment was calculated using the TP and FN predictions from the Big Data analytics based AI/ML protein conformation selection/prediction framework, described in Section 2.2. The base enrichment ratio is calculated to measure the effectiveness of general predictive performance in the absence of the ML protein conformation selection framework as in our prior work (Gupta et al., 2022). For accurate base enrichment ratio we performed subset data selection on previously calculated and published anticipated protein:ligand interactions energies in (Evangelista et al., 2016). The assumption is that the computed protein:ligand interaction energies are quantitatively valid, i.e., a “preferred” binding conformation would be the one in which the protein binds the ligand stronger (i.e., with lower interaction energies) than other alternative conformations. Thus, the base enrichment was calculated from (Evangelista et al., 2016) by dividing the number of binding conformations by the total number of conformations. Eq. 10 calculates the base enrichment detected during the test phase if the ML algorithm is not implemented. We select different subsets of the TP and FN values in order to calculate the ML prediction framework enrichment ratios in Eq. 10. The values returned by both Eqs 10, 11 were then used to calculate the final enrichment ratio returned by each of the four filters (A,B,C,D) defined in Eq. 12.

$$Base\;enrichment\;ratio=\frac{number\;of\;binding\;conformations}{total\;number\;of\;conformations\;\left(binding\;and\;non-binding\right)\;}$$ (10)

$$ML\;enrichment\;ratio=\frac{number\;of\;binding\;conformations\;\left(TP\right)\;identified}{number\;of\;total\;conformations\;\left(TP\;and\;FN\right)\;identified}$$ (11)

$$Final\;enrichment\;ratio=\frac{ML\;enrichment\;ratio}{Base\;enrichment\;ratio}$$ (12)

The final enrichment ratios for proteins ADORA2A, ADRB2, OPRD1, and OPRK1 were calculated using four different filters (A,B,C,D) and have been described and published in our previous work (Gupta et al., 2022). The proposed enrichment ratio framework used is depicted in Supplementary Figure SI-1 (Gupta et al., 2022).

### 2.2 The proposed Big Data analytics based AI/ML protein conformation selection/prediction framework

**FIGURE 3:**
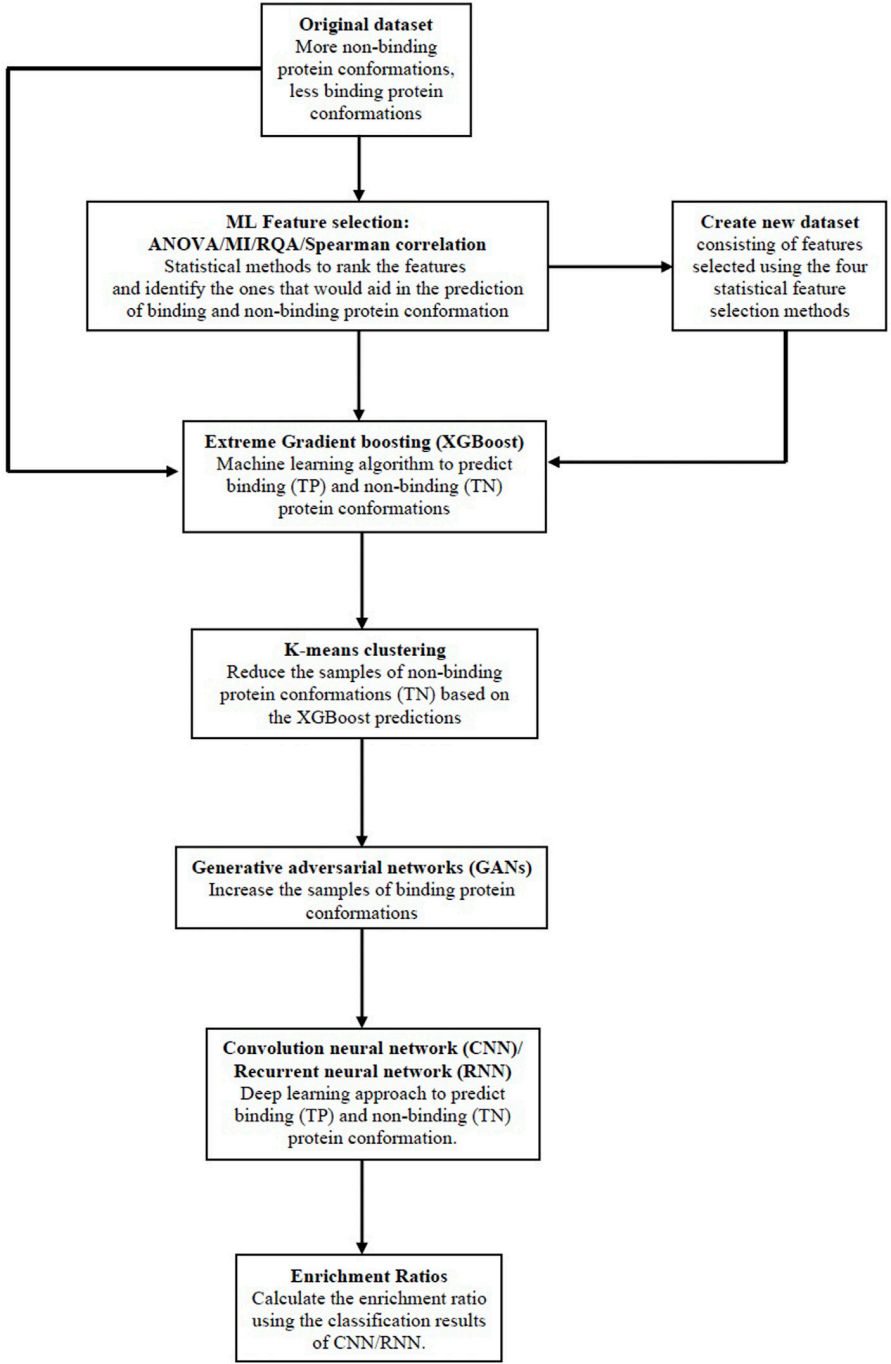
The proposed Big Data analytics based AI/ML protein conformation selection/prediction framework.

In this work, we combine the feature selection techniques discussed in Section 2.1 with the improved two-stage sampling based classification approach (Gupta et al., 2022) using deep learning techniques. The steps given below describe the new improved methodology and is illustrated in Figure 3:

• The first step in the methodology is to input the dataset and then apply the ML feature selection methods: i) Analysis of variance (ANOVA), ii) Mutual Information (MI), iii) Recurrence Quantification Analysis (RQA), and iv) Spearman correlation to select the important protein features from each of the methods respectively.
• We then obtain a feature ranking score for all features based on the common consensus of all the feature selection methods. Only the subset of protein features that are selected by all four feature selection methods are chosen to create a new dataset.
• Both the original dataset and a new dataset that is more biased towards samples in class 0 are sent as inputs to the XGBoost classifier. Samples of class 0 (TN) and class 1 (TP) are recorded as classification results 1.
• In order to create a new training dataset, the GAN algorithm was applied to both the original dataset and the new modified dataset.
• K-Means clustering (Akondi et al., 2019) is used on the XGBoost classifier’s classification results, class 0 samples are undersampled, and the intended class 1 samples are oversampled. This step increases the detection rate of class 1 samples or binding protein conformations to address the class imbalance issue. The new training dataset has the same size as the initial training dataset in order to maintain consistency.
• Supervised classification using deep learning methodologies: CNN and RNN are applied to the newly created training dataset. Both classifiers are used to identify the binding and non-binding conformations in the new training dataset. The results of both classifiers are recorded.
• As a final step, the TP (binding conformations), and FN (binding conformations but are incorrectly predicted as non-binding conformations) by the AI/ML protein conformation prediction framework (CNN and RNN) are employed in the Enrichment ratio framework to calculate the Enrichment ratios. The outcomes of the framework for enrichment ratios are recorded.

## 3 Results

The overview of enrichment ratios for ADORA2A that were determined using the predicted binding conformations from the AI/ML framework is shown in Table 1. As indicated in Supplementary Table SI-1 through Supplementary Table SI-5, the AI/ML framework was evaluated on the remaining 70% of the dataset after being trained on 30% of it. It can be observed that the data selection filter A of the Enrichment ratio framework gave the maximum enrichment ratio of 7.1 using XGBoost + GANs–RNN framework predictions.

**TABLE 1:** Enrichment Ratios of ADORA2A on the original dataset with no feature selection with training size of 30%.

Classifier	Maxima	Filter	% of data	Minima	Filter	% of data
XGboost + GANs–CNN	5.6	Filter A	0.5	4.1	Filter C	1.0
XGboost + GANs–RNN	7.1	Filter A	1.0	5.9	Filter C	0.5

The list of protein descriptors that the four ML feature selection techniques determined to be significant is shown in Table 2 and it can be observed that 11 of the 50 features were chosen. Table 3 gives the overview of the enrichment ratios that were calculated using the features listed in Table 2. It can be observed that data selection filter A of Enrichment ratio framework gave the maximum enrichment ratio of 10.2 using XGBoost + GANs–CNN framework predictions.

**TABLE 2:** 11 features out of 50 were selected having a feature score of four using the feature scoring table for ADORA2A.

pro_asa_vdw	pro_dipole_moment	pro_patch_ion_n	pro_patch_neg_n
pro_asa_hyd	pro_hyd_moment	pro_app_charge	pro_zquadrupole
pro_volume	pro_patch_ion	pro_helicity	

**TABLE 3:** Enrichment Ratios of ADORA2A on the dataset consisting of features as shown in Table 2 with training size of 30%.

Classifier	Maxima	Filter	% of data	Minima	Filter	% of data
XGboost + GANs–CNN	10.2	Filter A	0.5	8.1	Filter C	10.0
XGboost + GANs–RNN	9.0	Filter B	0.5	6.5	Filter D	1.0

The three common protein descriptors for the proteins ADORA2A, OPRK1, and OPRD1 that were determined to be significant by the four ML feature selection methods are listed in Supplementary Table SI-6. A summary of the enrichment ratios that were estimated using the characteristics indicated in Supplementary Table SI-6 is provided in Supplementary Table SI-7. It can be observed that employing data selection filter A, the XGBoost + GANs–CNN framework predictions provided the maximum enrichment ratio of 8.2.

The overview of enrichment ratios for the ADRB2 binding conformations predicted by the AI/ML framework is shown in Table 4. On 30% of the dataset, the AI/ML framework was trained, and on the remaining 70%, it was tested. It can be observed that employing data selection filter C, the XGBoost + GANs–RNN framework predictions provided the maximum enrichment ratio of 13.8.

**TABLE 4:** Enrichment Ratios of ADRB2 on the original dataset with no feature selection with training size of 30%.

Classifier	Maxima	Filter	% of data	Minima	Filter	% of data
XGboost + GANs–CNN	9.4	Filter C	10.0	6.7	Filter B	0.5
XGboost + GANs–RNN	13.8	Filter C	1.0	7.6	Filter A	1.0

The list of protein descriptors that the three out of four ML feature selection techniques determined to be significant is shown in Table 5. It can be seen from the table that 8 of the 51 features were chosen. Table 6 provides an overview of the enrichment ratios that were computed using the features listed in Table 5. It can be observed that employing data selection filter D, the XGBoost + GANs–RNN framework predictions provided the maximum enrichment ratio of 24.2.

**TABLE 5:** 8 features out of 51 were selected having a feature score of three using the feature scoring table for ADRB2.

pro_dipole_moment	pro_patch_hyd_5	pro_patch_pos_2
pro_patch_hyd	pro_patch_neg	pro_patch_hyd_1
pro_patch_hyd_4	pro_patch_neg_1	

**TABLE 6:** Enrichment Ratios of ADRB2 on the dataset consisting of features as shown in Table 5 with training size of 30%.

Classifier	Maxima	Filter	% of data	Minima	Filter	% of data
XGboost + GANs–CNN	18.1	Filter D	10.0	8.4	Filter A	0.5
XGboost + GANs–RNN	24.2	Filter D	10.0	13.5	Filter B	1.0

The summary of enrichment ratios for OPRD1 that were determined using the predicted binding conformations from the AI/ML framework is shown in Table 7. On 30% of the dataset, the AI/ML framework was trained, and on the remaining 70%, it was tested. It can be observed that utilizing data selection filter B, the XGBoost + GANs–RNN framework predictions produced an enrichment ratio of up to 37.

**TABLE 7:** Enrichment Ratios of OPRD1 on the original dataset with no feature selection with training size of 30%.

Classifier	Maxima	Filter	% of data	Minima	Filter	% of data
XGboost + GANs–CNN	12.5	Filter B	0.5	4.9	Filter C	10.0
XGboost + GANs–RNN	37.5	Filter B	0.5	27.6	Filter D	5.0

The list of protein descriptors that the four ML feature selection techniques determined to be significant is shown in Table 8. It can be seen that 12 of the 51 features were chosen, and Table 9 provides an overview of the enrichment ratios that were computed using the features listed in Table 8. It can be observed that employing data selection filter B, the XGBoost + GANs–RNN framework predictions provided the maximum enrichment ratio of 37.5.

**TABLE 8:** 12 features out of 51 were selected having a feature score of four using the feature scoring table for OPRD1.

pro_asa_vdw	pro_hyd_moment	pro_patch_hyd_5	pro_patch_pos
pro_asa_hyd	pro_patch_hyd	pro_patch_neg	pro_net_charge
pro_asa_hph	pro_patch_hyd_4	pro_patch_neg_5	pro_app_charge

**TABLE 9:** Enrichment Ratios of OPRD1 on the dataset consisting of features as shown in Table 8 with training size of 30%.

Classifier	Maxima	Filter	% of data	Minima	Filter	% of data
XGboost + GANs–CNN	16.5	Filter B	1.0	11.2	Filter A	0.5
XGboost + GANs–RNN	37.5	Filter B	0.5	25.7	Filter D	0.5

Supplementary Table SI-8 gives the overview of enrichment ratios that were calculated using the features that were listed in Supplementary Table SI-6. It can be seen that both XGBoost + GANs–CNN and XGBoost + GANs–RNN framework predictions gave the same enrichment ratio of 37.5, using data selection filter B.

A summary of the enrichment ratios for OPRK1 that were determined using the predicted binding conformations from the AI/ML framework is shown in Table 10. On 30% of the dataset, the AI/ML framework was trained, and on the remaining 70%, it was tested. It can be seen that employing data selection filter A, the XGBoost + GANs–RNN framework predictions provided the maximum enrichment ratio of 27.6.

**TABLE 10:** Enrichment Ratios of OPRK1 on the original dataset with no feature selection with training size of 30%.

Classifier	Maxima	Filter	% of data	Minima	Filter	% of data
XGboost + GANs–CNN	11.0	Filter A	10.0	6.9	Filter B	0.5
XGboost + GANs–RNN	27.6	Filter A	1.0	21.0	Filter D	0.5

The list of protein descriptors that the four ML feature selection techniques determined to be significant is shown in Table 11. It can be seen that 5 of the 50 features were chosen, and Table 12 provides an overview of the enrichment ratios that were computed using the features listed in Table 11. It can be seen that both XGBoost + GANs–CNN and XGBoost + GANs–RNN frameworks gave the same enrichment ratio of 27.6, using data selection filter A.

**TABLE 11:** 5 features out of 50 were selected having a feature score of four using the feature scoring table for OPRK1.

pro_asa_vdw	pro_hyd_moment	pro_patch_neg_1
pro_asa_hyd	pro_patch_hyd_5	

**TABLE 12:** Enrichment Ratios of OPRK1 on the dataset consisting of features as shown in Table 11 with training size of 30%.

Classifier	Maxima	Filter	% of data	Minima	Filter	% of data
XGboost + GANs–CNN	27.6	Filter A	1.0	20.2	Filter D	0.5
XGboost + GANs–RNN	27.6	Filter A	1.0	21.0	Filter D	0.5

An overview of the enrichment ratios that were calculated using the descriptors listed in Supplementary Table SI-6 is provided in Supplementary Table SI-9. It can be seen that employing data selection filter A, the XGBoost + GANs–RNN framework predictions provided the maximum enrichment ratio of 30.1.

## 4 Discussion

The Big Data analytics research outcomes in this study suggest that four proteins ADORA2A, ADRB2, OPRK1, and OPRD1, and their binding conformations considered in this work do possess similar global properties that can be leveraged to predict whether they will be more likely to bind their ligands than other conformations. The enrichment factors obtained with the best approaches are about 10 to about 40 times better than what would be available with a random selection of protein conformations for docking. For three out of the four targets of interest here (i.e., ADORA2A, OPRK1, and OPRD1), the physico-chemical features that are most associated with a high propensity to be selected for binding by the ligands are the water accessible surface area (MOE descriptor *pro_asa_vdw*), the hydrophobic surface area (MOE descriptor *pro_asa_hyd*) and the hydrophobicity moment (MOE descriptor *pro_hyd_moment)*. That these properties, which are global and not limited to the binding sites, are common to the important descriptor of all proteins point to a dual role of exposure to solvent and hydrophobicity as globally driving the capacity of proteins to bind, or not, their ligands. Note that this work is not a structure-activity relationship studies, i.e., we do not at this point give a range of values for these proteins that would be associated with ligand binding and a range of values that would be associated with non-ligand binding.

The fourth protein target that was used here, ADRB2, can also be analyzed by deep learning approaches to identify the ligand binding conformations about 24 times better than a random selection of conformations. Yet, that one protein target yields different physico-chemical features than the other three proteins used here, although the general role of surface hydrophobicity and electrostatics (negatively-charged regions, precisely) is conserved. We do not yet know if this difference observed between ADRB2 and the other proteins is a result of different actual physicochemical mechanisms involved in ligand binding, or if this is an artifact of the data and of specific issues with class imbalance from the MD trajectories of this target.

Nonetheless, the fact that the apo-proteins’ global physico chemicals properties may—to an extent—predict the ligand-binding character of conformations is remarkable. Naturally, this does not mean that only global protein properties are “holding” the keys to the conformational selection mechanisms. This work will have to be continued and repeated with features that are specific to the binding sites’ conformations rather than describing the global protein structure.

## Data Availability

The datasets presented in this study can be found in online repositories. The names of the repository/repositories and accession number(s) can be found in the article/Supplementary Material.
