# Supplementary material for: Big Data analytics for improved prediction of ligand binding and conformational selection
Source: Front Mol Biosci. 2023 Jan 12;9:953984. doi: 10.3389/fmolb.2022.953984 (PMC9878559; doi:10.3389/fmolb.2022.953984)
Supplement: Supplementary file 1 [file Table1.DOCX]

***Supplementary Material***

# Supplementary Tables

**Table SI-1.** Samples in the Training and testing phase of the ML framework for each protein

| **Protein** | Class 0 (number of non-binding conformations) samples | | Class 1 (number of binding conformations) samples | |
| --- | --- | --- | --- | --- |
|  | ***Training*** | ***Testing*** | ***Training*** | ***Testing*** |
| **ADORA2A** | 630 | 1517 | 268 | 583 |
| **ADRB2** | 719 | 1692 | 50 | 104 |
| **OPRD1** | 883 | 2049 | 18 | 54 |
| **OPRK1** | 858 | 2004 | 41 | 96 |

**Table SI-2.** Classification table of ADORA2A with a training size of 30%

| **Dataset Type** | | **Classifier** | **TP** | **FN** | **Accuracy(%)** | **Sensitivity(%)** |
| --- | --- | --- | --- | --- | --- | --- |
| **Original Dataset** | | XGBoost+GANs-CNN | 209 | 374 | 61 | 35.85 |
|  |  | XGBoost+GANs-RNN | 253 | 330 | 54.29 | 43.40 |
| **Dataset consisting of features as shown in table 2** | | XGBoost+GANs-CNN | 302 | 281 | 50.48 | 51.8 |
|  |  | XGBoost+GANs-RNN | 250 | 333 | 51.7 | 42.9 |
| **Dataset consisting of features as shown in table 4** | | XGBoost+GANs-CNN | 252 | 331 | 52.71 | 43.2 |
|  |  | XGBoost+GANs-RNN | 251 | 332 | 51.1 | 43.1 |

**Table SI-3.** Classification table of ADRB2 with a training size of 30%

| **Dataset Type** | | **Classifier** | **TP** | **FN** | **Accuracy(%)** | **Sensitivity(%)** |
| --- | --- | --- | --- | --- | --- | --- |
| **Original Dataset** | | XGBoost+GANs-CNN | 32 | 80 | 73.59 | 28.57 |
|  |  | XGBoost+GANs-RNN | 51 | 61 | 44.51 | 45.5 |
| **Dataset consisting of features as shown in table 7** | | XGBoost+GANs-CNN | 58 | 54 | 47.58 | 51.8 |
|  |  | XGBoost+GANs-RNN | 77 | 35 | 41.94 | 68.8 |

**Table SI-4.** Classification table of OPRD1 with a training size of 30%

| **Dataset Type** | | **Classifier** | **TP** | **FN** | **Accuracy(%)** | **Sensitivity(%)** |
| --- | --- | --- | --- | --- | --- | --- |
| **Original Dataset** | | XGBoost+GANs-CNN | 3 | 51 | 83.4 | 5.6 |
|  |  | XGBoost+GANs-RNN | 30 | 24 | 45.69 | 55.6 |
| **Dataset consisting of features as shown in table 10** | | XGBoost+GANs-CNN | 15 | 39 | 63.15 | 27.8 |
|  |  | XGBoost+GANs-RNN | 25 | 29 | 58.8 | 46.3 |
| **Dataset consisting of features as shown in table 4** | | XGBoost+GANs-CNN | 32 | 22 | 75.18 | 59.3 |
|  |  | XGBoost+GANs-RNN | 33 | 21 | 74.94 | 61.1 |

**Table SI-5.** Classification table of OPRK1 with a training size of 30%

| **Dataset Type** | | **Classifier** | **TP** | **FN** | **Accuracy(%)** | **Sensitivity(%)** |
| --- | --- | --- | --- | --- | --- | --- |
| **Original Dataset** | | XGBoost+GANs-CNN | 25 | 76 | 78.13 | 24.75 |
|  |  | XGBoost+GANs-RNN | 52 | 49 | 50.4 | 51.49 |
| **Dataset consisting of features as shown in table 14** | | XGBoost+GANs-CNN | 50 | 51 | 57.5 | 49.5 |
|  |  | XGBoost+GANs-RNN | 52 | 49 | 56.98 | 51.5 |
| **Dataset consisting of features as shown in table 4** | | XGBoost+GANs-CNN | 52 | 49 | 57.26 | 51.5 |
|  |  | XGBoost+GANs-RNN | 58 | 43 | 55.84 | 57.4 |

**Table SI-6.** 3 features out of 50 were selected that are common between proteins ADORA2A, ORPD1 and OPRK1.

| pro_asa_vdw | pro_asa_hyd | pro_hyd_moment |
| --- | --- | --- |

**Table SI-7.** Enrichment Ratios of ADORA2A on the dataset consisting of features as shown in table 5 with training size of 30%

| **Classifier** | **Maxima** | **Filter** | **% of data** | **Minima** | **Filter** | **% of data** |
| --- | --- | --- | --- | --- | --- | --- |
| XGboost + GANs - CNN | 8.2 | Filter A | 0.5% | 6.8 | Filter D | 1.0% |
| XGboost + GANs - RNN | 8.1 | Filter A | 0.5% | 6.6 | Filter D | 1.0% |

**Table SI-8.** Enrichment Ratios of OPRD1 on the dataset consisting of features as shown in table 5 with training size of 30%

| **Classifier** | **Maxima** | **Filter** | **% of data used** | **Minima** | **Filter** | **% of data used** |
| --- | --- | --- | --- | --- | --- | --- |
| XGboost + GANs - CNN | 37.5 | Filter B | 0.5% | 27.9 | Filter D | 5.0% |
| XGboost + GANs - RNN | 37.5 | Filter B | 0.5% | 28.2 | Filter D | 5.0% |

**Table SI-9.** Enrichment Ratios of OPRK1 on the dataset consisting of features as shown in table 5 with training size of 30%

| **Classifier** | **Maxima** | **Filter** | **% of data** | **Minima** | **Filter** | **% of data** |
| --- | --- | --- | --- | --- | --- | --- |
| XGboost + GANs - CNN | 27.6 | Filter A | 1.0% | 21.0 | Filter D | 0.5% |
| XGboost + GANs - RNN | 30.1 | Filter A | 1.0% | 22.9 | Filter D | 0.5% |

##

##

##

##

## 2. Supplementary Figures


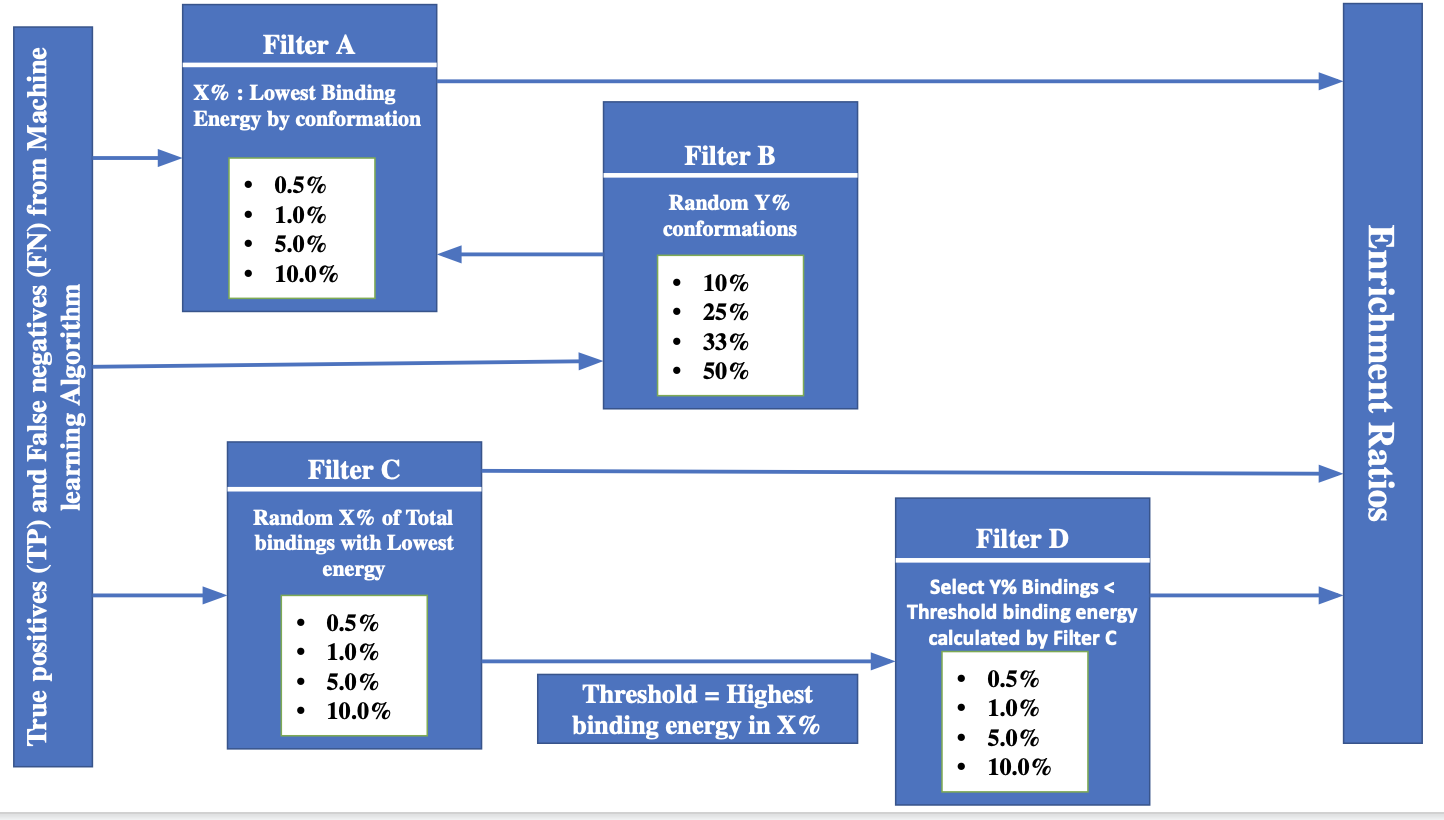


**Figure SI-1**: The proposed enrichment ratio framework [8]
