# Supplementary figures and images for: Big Data analytics for improved prediction of ligand binding and conformational selection
Source: Front Mol Biosci. 2023 Jan 12;9:953984. doi: 10.3389/fmolb.2022.953984 (PMC9878559; doi:10.3389/fmolb.2022.953984)

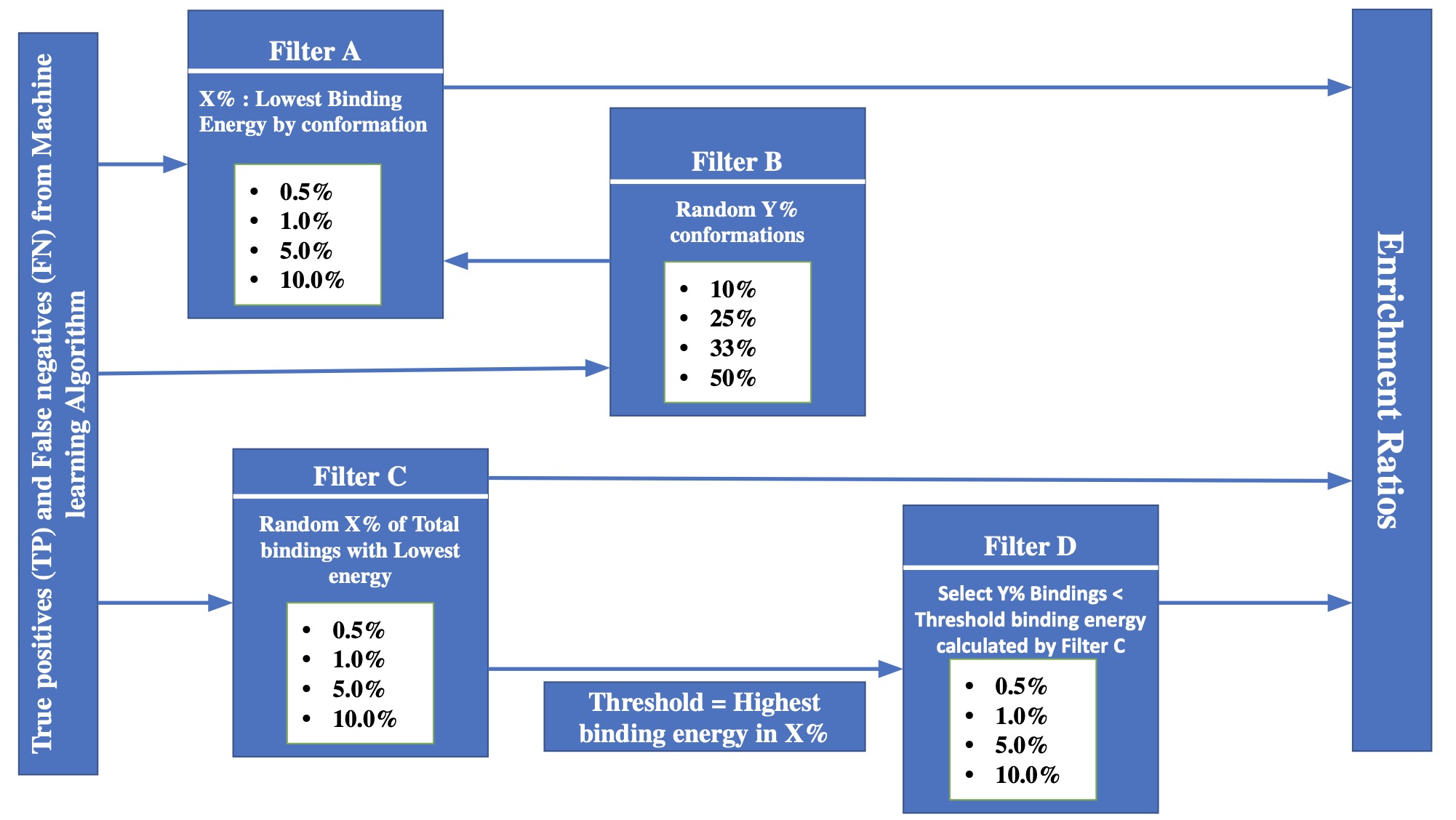

Supplement: Supplementary file 2 [file Image1.JPEG]
